# Recorded Behavior as a Valuable Resource for Diagnostics in Mobile Phone Addiction: Evidence from Psychoinformatics

**DOI:** 10.3390/bs5040434

**Published:** 2015-10-19

**Authors:** Christian Montag, Konrad Błaszkiewicz, Bernd Lachmann, Rayna Sariyska, Ionut Andone, Boris Trendafilov, Alexander Markowetz

**Affiliations:** 1 Institute of Psychology and Education, Ulm University, Ulm, 89081, Germany; E-Mails: bernd.lachmann@uni-ulm.de (B.L.); rayna.sariyska@uni-ulm.de (R.S.); 2 Department of Informatics, University of Bonn, Bonn, 53117, Germany; E-Mails: konrad.blaszkiewicz@gmail.com (K.B.); andone@informatik.uni-bonn.de (I.A.); trendafilov.boris@gmail.com (B.T.); alex@iai.uni-bonn.de (A.M.)

**Keywords:** computer science, psychology, diagnostics, behavioral addiction, psychoinformatics

## Abstract

Psychologists and psychiatrists commonly rely on self-reports or interviews to diagnose or treat behavioral addictions. The present study introduces a novel source of data: recordings of the actual problem behavior under investigation. A total of *N* = 58 participants were asked to fill in a questionnaire measuring problematic mobile phone behavior featuring several questions on weekly phone usage. After filling in the questionnaire, all participants received an application to be installed on their smartphones, which recorded their phone usage for five weeks. The analyses revealed that weekly phone usage in hours was overestimated; in contrast, numbers of call and text message related variables were underestimated. Importantly, several associations between actual usage and being addicted to mobile phones could be derived exclusively from the recorded behavior, but not from self-report variables. The study demonstrates the potential benefit to include methods of psychoinformatics in the diagnosis and treatment of problematic mobile phone use.

## 1. Introduction

Nowadays, among the most important communication channels are (i) the Internet, and (ii) smartphones, which of course can be also used as a gateway to the Internet. Globally, there is a growing debate as to whether an overuse of mobile phones and the Internet can be defined as a form of a behavioral addiction [1,2,3]. Overuse clearly cannot be understood simply by referring to the time spent on mobile phones or on the Internet, but rather other addiction-related concepts such as preoccupation, withdrawal, development of tolerance or personal suffering because of the usage are more important variables. The importance of the topic is reflected by the large number of conducted studies [4,5] and also by the inclusion of Internet Gaming Disorder as an emerging disorder in Section III of the DSM-5. More and more focus is also on smartphone addiction [6,7,8]. Smartphones represent a more sophisticated version of the overarching category of mobile phone. Given the controversy about the nature of smartphone overuse, “problematic mobile phone use” might be a better term compared to “mobile phone addiction” or “smartphone addiction”. However, given the handiness of the term “addiction”, we use both the terms “problematic use” and “addiction” synonymously in the present manuscript. A further note: Although the terms “mobile phone” and “smartphone” are not exactly the same (as outlined above), for reasons of simplicity and also because of the fact that we study classic variables such as call and SMS behavior, the terms are used somewhat interchangeably in the present study. It is worth noting that we did not study usage of social messenger channels such as Facebook or WhatsApp in the present research endeavor.

In a recent paper, we outlined the need to include methods from computer science in diagnostics to get a fuller picture of psychopathological disorders [9]. In that paper, we also coined the term “psychoinformatics” describing the administration of computer science methods (such as tracking smartphone behavior) to study psychological phenotypes. The same term has also been introduced by Yarkoni [10]. Not only could diagnostics of behavioral addictions being related to technology (e.g., excessive online video gaming, online gambling, mobile phone usage) benefit from the inclusion of actual recorded behavior, but so too could the course of therapy of these non-substance related addictions [11]. Additionally, mobile phone applications can also be of value in the treatment (e.g., aftercare) of addictions such as alcoholism [12].

Naturally, actual behavior should constitute a better predictor for addictive tendencies than self-reported variables. As attractive as this idea seems, only some recent studies could in parts back up this idea [13,14]. The present study aims to find further support, while investigating excessive mobile phone behavior (with a focus on smartphones). We compared self-reported data with directly recorded data over several mobile phone variables (including calls and SMS) to predict mobile phone addiction. It has earlier been shown that recorded behavior differs from self-reported data on mobile phone usage [13,14]. Beyond this research question, we asked ourselves if actual recorded mobile phone behavior in hours is more closely related to self-reported addictive mobile phone usage tendencies compared to self-assessed mobile phone behavior in hours.

First, we hypothesized that participants would have problems assessing their mobile phone usage, thereby (according to [14]) underestimating their actual use. Second, we hypothesized that actual recorded mobile phone behavior compared to the self-reported assessments should be more closely associated with results from the mobile phone addiction questionnaire.

## 2. Materials & Methods

### 2.1. Participants

A total of *N* = 58 healthy participants (33 males and 25 females) with a mean age of 24.22 (SD = 5.02, age-range = 18–46) participated in the present study. Participants were recruited via classes in psychology and computer science. After participation all participants got a monetary compensation. All participants came to our laboratory and received the self-developed application called Menthal on their phones. This software records mobile phone behavior such as number of incoming/outgoing calls or length of app usage. Importantly, and in contrast to the publicly available version of Menthal, the version used in this study presented no feedback to the user. Besides the complete length of phone usage, it recorded call- and SMS-related variables for five weeks and computed means for average use of the variables on a weekly basis (see also Table 1). In order to be able to compare these recorded smartphone variables with the self-reported data, we asked the participants to assess these variables before getting the application installed on their mobile phones (in the same session when the app was installed). Participants were asked to provide us with the average mobile phone behavior for a week, in the context of the mentioned mobile phone variables. A subsample (*n* = 49) of the participants was investigated in the context of smartphone behavior and personality in an earlier study [15]. The study was approved by the local ethics committee of the University of Bonn, Germany. All participants gave written consent.

**Table 1:** Comparison of recorded and self-reported means for phone related variables.

Variables of Interest Paired in Self-Report *vs.* Recorded	Mean and Standard Devitation (Median and Interquartile Range)	Significance Test (Wilcoxon Signed Rank Test)
Self-reported weekly phone use (*N* = 58)	15.79 (SD = 18.04) (10.00 (IR = 13.50))	*z* = −1.90, *p* = 0.06
Recorded weekly phone use (*N* = 58)	10.16 (SD = 6.06) (9.51 (IR = 8.65))	
Self-reported incoming calls each week (*N* = 58)	3.83 (SD = 3.62) (3.00 (IR = 4.00))	*z* = −4.43, *p* < 0.01 *
Recorded incoming calls each week (*N* = 58)	6.55 (SD = 5.10) (5.25 (IR = 6.14))	
Self-reported outgoing calls each week (*N* = 58)	3.72 (SD = 3.70) (2.75 (IR = 3.25))	*z* = −6.45, *p* < 0.01 *
Recorded outgoing calls each week (*N* = 58)	14.31 (SD = 10.35) (10.75 (IR = 13.53))	
Self-reported incoming SMS each week (*N* = 58)	9.30 (SD = 6.12) (9.00 (IR = 8.75))	*z* = −3.79, *p* < 0.01 *
Recorded incoming SMS each week (*N* = 58)	24.05 (SD = 27.52) (16.28 (IR = 20.46))	
Self-reported outgoing SMS each week (*N* = 44)	10.72 (SD = 9.86) (9.00 (IR = 10.75)	*z* = −0.76, *p* = 0.22 ^+^
Recorded outgoing SMS each week (*N* = 58)	17.83 (SD = 26.37) (10.26 (IR = 16.29)	
Recorded outgoing SMS each week (*N* = 44)	18.40 (SD = 26.38) (11.24 (IR = 16.12)	

Note: * holds for multiple testing after Bonferroni adjustment (α = 0.05 divided by five tests = α = 0.01); ^+^ as the Wilcoxon signed rank test requires same sample sizes we built a second group for the last variable consisting out *N* = 44 instead of *N* = 58.

### 2.2. Questionnaire to Assess (Problematic) Use of the Mobile Phone

Along with items related to the use of different facets of mobile phones we administered our own translated German version of the Mobile Phone Problem Use Scale (MPPUS) [1] to assess tendencies for excessive mobile phone use. The MPPUS measures problematic use of mobile phones along 27 items. The content of the items deals with several mobile phone topics, including control issues over the mobile phone, bodily pains and everyday problems due to the mobile phone usage. For further clarity, here is an example item of the MMPUS: “I find it difficult to switch off my mobile phone”. (Item 13, p. 43 in [1]). The present study administered the MPPUS with a five point Likert scale (“not true at all” to “extremely true”). Therefore, scores ranged between 27 and 135. Higher scores indicated more serious problems due to mobile phone usage. Of note, the original questionnaire uses a 10 point Likert scale with the same poles as mentioned above. As the German version of the MPPUS is also used in other contexts in our work group, we decided, for comparability reasons, to use a five point Likert scale. Internal consistency of the MPPUS of the present German version (self-translated) was α = 0.86. Finally, we would like to add the following information: As already outlined, mobile phones can be seen as a broad category of phones carried on the body to communicate. Smartphones represent a subcategory and are characterized by sophisticated features going beyond calling and writing short text messages. The MPPUS was developed at a time when smartphones were not yet available. By filling in this questionnaire while owning a smartphone (as all participants of the study did), it is clear that the questionnaire deals with smartphone addiction.

### 2.3. Mobile Phone Application (Menthal)

Menthal was installed on the phone of every participant by members of our own work group. This application collects data on many events. For example, it tracks when the screen was switched on, is unlocked, or when an application is started. All events are associated with the user-ID and a timestamp. Similarly, the software tracks phone calls and text messages by accessing the phone’s log records. All the data were securely stored on the phone, and transferred to our server via an encrypted connection. The raw data events were then transformed into phone sessions, each indicating an uninterrupted interval of interaction with the phone. Derived from raw data events, each phone session on the server was converted to the following format: User-ID, start-time, stop-time. As we were only interested in active interaction (*i.e.*, the user unlocked the phone and was physically interacting with the visual interface), no phone session was generated, when, for example, the user was using his phone as music player while the screen was locked. The variables of interest in the present study are presented in Table 1.

### 2.4. Statistical Analyses

As all dependent variables of interest deviated from normal distribution (clarified by visual inspection of the data), Wilcoxon rank sum tests were computed to compare self-reported data with actual recorded data. Mann-Whitney-U tests were used for evaluating the effects of gender on the mobile phone variables. Spearman’s correlations were computed to assess the link between both kinds of mobile phone variables and the self-report questionnaire MPPUS assessing mobile phone addiction. In addition to these analyses, we conducted Fisher’s Z test to characterize the statistical differences between the observed correlation patterns. We present results for two-sided testing below.

## 3. Results

### 3.1. Age, Gender and the Variables of Investigation

Age correlated negatively with the MPPUS score (ρ = −0.32, *p* = 0.02). The same was true for age and SMS sent out weekly (recorded; ρ = −0.34, *p* = 0.008). Additionally, incoming calls each week (recorded: ρ = 0.27, *p* = 0.04) were associated with age, but this time the correlation was positive. Gender significantly influenced one of the SMS related variables with females being associated with more recorded incoming SMS (U(33,25) = 249.00, *p* = 0.01) and outgoing SMS (U(33,25) = 266, *p* = 0.02). The same was true for the MPPUS score (U(33,25) = 271.50, *p* = 0.03) and general phone usage in terms of self-report (U(33,25) = 257.50, *p* = 0.02). As the age correlations and gender differences were not hypothesized, we did not include these variables for further analyses. Moreover, given our rather small sample size, the analyses of age and gender are not overtly meaningful.

### 3.2. Self-Reported vs. Recorded Behavior of Mobile Phone Use: A Comparison

Table 1 presents the means and standard deviations of the investigated variables of interest. Moreover, we report median and interquartile ranges. The results of the Wilcoxon rank sum tests are presented in the last column of this table. The results can be summarized as follows: total phone usage was overestimated by the participants, whereas more distinct variables of smartphone behavior were underestimated. An exception was the contrast of self-reported *vs.* recorded number of outgoing SMS, failing by far to reach significance. This might be due to the smaller sample—14 participants did not fill in this item. The remaining variables have been recorded and filled in completely.

### 3.3. Correlating Mobile Phone Addiction Data from Self-Report vs. Actual Recorded Mobile Phone Addiction

Table 2 illustrates that the correlations between the MMPUS questionnaire and length of weekly phone usage (assessed via recording or self-report) reach about the same level. In contrast, two associations between directly recorded SMS variables (number of incoming/outgoing SMS) appeared with the MMPUS, which could not be observed with the self-reported data. It is important to note that only Fisher’s Z test for the contrast “number of outgoing SMS” and MPPUS scores for the self-reported and recorded variables was significant.

**Table 2:** Correlations between Mobile Phone Problem Use Scale (MPPUS) and recorded telephone variables; correlations are presented with *p*-values for two-sided tests.

	Phone Use Recorded in Hours a Week	Number of Incoming Calls	Number of Outgoing Calls	Number of Incoming SMS	Number of Outgoing SMS
Self-report MPPUS (rho)	ρ = 0.41, *p* = 0.001 * (*N* = 58)	ρ = 0.03, *p* = 0.80 (*N* = 58)	ρ = −0.01, *p* = 0.94 (*N* = 58)	ρ = 0.10, *p* = 0.45 (*N* = 58)	ρ = 0.04, *p* = 0.79 (*N* = 44)
Recorded MPPUS (rho)	ρ = 0.37, *p* = 0.004 * (*N* = 58)	ρ = 0.04, *p* = 0.79 (*N* = 58)	ρ = 0.24, *p* = 0.08 (*N* = 58)	ρ = 0.36, *p* = 0.006 * (*N* = 58)	ρ = 0.42, *p* = 0.001 * (*N* = 58)
Results from Fisher’s Z-Test	*z* = 0.25 *p* = 0.80	*z* = −0.05 *p* = 0.96	*z* = −1.34 *p* = 0.18 ^+^	*z* = −1.45 *p* = 0.15 ^+^	*z* = −1.98 *p* = 0.047

Note: * holds for multiple testing after Bonferroni adjustment (α = 0.05 divided by five tests = α = 0.01); ^+^ indicates that, arguably, one could have presented *p*-values for one-sided testing resulting in trend significant results.

## 4. Discussion

The present study compared relevant variables for problematic mobile phone usage, as recorded directly on the phone, with self-reported assessments from the users. To some degree in line with our first hypothesis, we observed that the aggregated length of weekly mobile phone usage in hours was overestimated by the participants of the study, while more distinct behaviors, such as weekly outgoing calls, have been underestimated. Of note, the overestimation effect would have been significant if results from a one-sided test procedure were presented (or arguably with a larger sample size). These summarized results indicate that the participants were not able to estimate their mobile phone usage in correct numbers. Our study results differ in parts from those presented by Lin *et al.* [14], who only reported underestimation of smartphone usage in their study. This could be due to differences in questions asked of participants. According to Lin *et al.* (2015), they asked “participants how many hours [they spent] on their smartphone on average during a weekday, and then asked if there was any difference between their weekday and weekend use” [14] (p. 141). In contrast we asked our participants simply how much they estimated their weekly usage to be in hours. Furthermore, we also addressed more specific activities such as incoming calls. Moreover, awareness of the topic of study might have led to an exaggerated number of total mobile phone usage in our study. However, this might also be true for the Lin *et al.* study. Finally, in the Lin *et al.* study a substantial part of the sample was characterized as being smartphone addicted (31 out of 79 participants), whereas our sample seemed to be largely in the normal usage range. For further illustration: Mean of the MPPUS was 54.47 (SD = 14.45); median = 51.50 (IR = 20.75)—please remember that scores range between 27 and 135 (our data set ranged from 30–89).

In line with our second hypothesis, the overall pattern of correlations between the recorded and self-reported variables and the mobile phone addiction scores demonstrates that the recorded behavior is more strongly associated with addictive tendencies—two out of five associations would have not been found when only asking the participants. These findings illustrate the potential benefits from close collaborations between computer science and psychiatry/psychology, which would allow the inclusion of direct tracking of behavior. These methods could aid the diagnostic process and therapy by objectively recording the behavioral addiction of interest [11].

We are convinced that the present results can be transferred to some extent to other technological behavioral addictions such as Internet addiction. We come to this conclusion because smartphone addiction and Internet addiction overlap as outlined by Kwon [6,7]. Moreover, both forms of addiction rely on technology use, which can be recorded. As outlined by others [14], users of electronic devices seem to have problems assessing the time they spend online. Psychoinformatics will help them to get exact feedback on the actual media consumption. How psychoinformatics will be of relevance for diagnostics and treatment of substance-related addictions is an important question for further research. Here, text mining, the statistical analyses of text in SMS and emails, can reveal information about the mood of a person (by observing and counting the use of negative and positive words). Although this might be a great leap from the present results, insights into emotional states are of high importance when studying addiction (e.g., withdrawal goes along with negative emotionality). Other functions such as GPS tracking will be able to give new insights into addiction such as that a very stressful life in terms of excessive mobility might be associated with other forms of addiction, even substance related addictions like higher alcohol consumption. (e.g., Internet addiction has repeatedly been associated with higher alcohol consumption) [16,17].

At this point, we want to discuss a number of limitations of the present study. In the current study, we decided to look at classical smartphone variables exclusively (primarily to avoid the problem of multiple tests when exploring numerous variables). However, a future study will need to monitor activities in social networks or other instant messengers such as WhatsApp, which are currently heavily consumed by users and might be even more strongly associated with mobile phone and smartphone addiction compared to the present variables of interest. In this context, we refer to a new study [18] showing that WhatsApp usage is one of the driving forces of smartphone usage. Additionally, the present study investigated a rather small number of participants stemming from university campuses (thus, making it hard to take gender and age into account as control variables). As well, the investigation of clinical samples in the context of addictive or problematic behavior clearly is also warranted. Consequently, the results need to be replicated in more representative samples or participants at stronger risk for the problem behavior under investigation. Other variables such as number of logins on the smartphone (and hence the fragmentation of everyday life) might be also very interesting. Finally, we discarded the first week of data in order to tackle the problem that the recorded behavior of the present study might have been influenced by feelings of being observed. This approach was based on no empirical evidence and future studies need to provide numbers to deal with this problem more adequately.

Nevertheless we are of the opinion that the empirical data of this study represent an important (although preliminary) starting point to illustrate the advantages of including computer science methods in psychology and psychiatry.

However, even after taking into account some of the advantages of objectively recorded data, one can imagine that psychology and psychiatry would not be possible without self-report data. In our opinion, self-report data was and will always be an important data source when dealing with well-being or the mental state of a person. In this context, we hold that a combination of self-report data and actual recording of the problem behavior will provide the clearest picture of the patient, as well as giving insight into to the ability of the patient to reflect about his or her addiction. This is underlined by some data in Table 1: the sometimes differing means/standard deviations (either high or low) show that self-assessment and actual behavior clearly are not the same.

## 5. Conclusions

Many opportunities arise from the use of new technologies and digital methods with psychology and psychiatry. Of course, dangers of misuse are legion. However, the well-established principle of strict confidentiality between psychologists/psychiatrists and patients will provide a good framework for the use of this new important data pool. This important topic cannot be discussed in this brief communication but is commented on in our recent theoretical paper on diagnostics [9]. Concluding, psychoinformatics reflect a new promising tool for assessing and treating mobile phone addiction.
